# Spatial and single-cell analyses uncover links between ALKBH1 and tumor-associated macrophages in gastric cancer

**DOI:** 10.1186/s12935-024-03232-5

**Published:** 2024-02-06

**Authors:** Renin Chang, Kuan-Hao Tsui, Li-Fei Pan, Chia-Jung Li

**Affiliations:** 1 Department of Emergency Medicine, Kaohsiung Veterans General Hospital, Kaohsiung, Taiwan; 2 Department of Recreation and Sports Management, Tajen University, Pingtung, Taiwan; 3 Institute of Medicine, Chung Shan Medical University, Taichung, Taiwan; 4 Department of Obstetrics and Gynaecology, Kaohsiung Veterans General Hospital, Kaohsiung, Taiwan; 5 Institute of BioPharmaceutical Sciences, National Sun Yat-Sen University, Kaohsiung, Taiwan; 6 Department of Obstetrics and Gynaecology, National Yang-Ming University School of Medicine, Taipei, Taiwan; 7 Department of Medicine, Tri-Service General Hospital, National Defense Medical Center, Taipei, 114 Taiwan; 8 Department of General Affair Office, Kaohsiung Veterans General Hospital, Kaohsiung, 813 Taiwan

**Keywords:** ALKBH1, Immune infiltration, Gastric cancer, Single-cell RNA sequencing, Spatial transcriptomics

## Abstract

**Background:**

AlkB homolog 1, histone H2A dioxygenase (ALKBH1), a crucial enzyme involved in RNA demethylation in humans, plays a significant role in various cellular processes. While its role in tumor progression is well-established, its specific contribution to stomach adenocarcinoma (STAD) remains elusive. This study seeks to explore the clinical and pathological relevance of ALKBH1, its impact on the tumor immune microenvironment, and its potential for precision oncology in STAD.

**Methods:**

We adopted a comprehensive multi-omics approach to identify ALKBH1 as an potential diagnostic biomarker for STAD, demonstrating its association with advanced clinical stages and reduced overall survival rates. Our analysis involved the utilization of publicly available datasets from GEO and TCGA. We identified differentially expressed genes in STAD and scrutinized their relationships with immune gene expression, overall survival, tumor stage, gene mutation profiles, and infiltrating immune cells. Moreover, we employed spatial transcriptomics to investigate ALKBH1 expression across distinct regions of STAD. Additionally, we conducted spatial transcriptomic and single-cell RNA-sequencing analyses to elucidate the correlation between ALKBH1 expression and immune cell populations. Our findings were validated through immunohistochemistry and bioinformatics on 60 STAD patient samples.

**Results:**

Our study unveiled crucial gene regulators in STAD linked with genetic variations, deletions, and the tumor microenvironment. Mutations in these regulators demonstrated a positive association with distinct immune cell populations across six immune datasets, exerting a substantial influence on immune cell infiltration in STAD. Furthermore, we established a connection between elevated ALKBH1 expression and macrophage infiltration in STAD. Pharmacogenomic analysis of gastric cancer cell lines further indicated that ALKBH1 inactivation correlated with heightened sensitivity to specific small-molecule drugs.

**Conclusion:**

In conclusion, our study highlights the potential role of ALKBH1 alterations in the advancement of STAD, shedding light on novel diagnostic and prognostic applications of ALKBH1 in this context. We underscore the significance of ALKBH1 within the tumor immune microenvironment, suggesting its utility as a precision medicine tool and for drug screening in the management of STAD.

## Introduction

STAD predominantly affects populations in Asian and South American countries [1], and is characterized by high levels of intra- and inter-tumor heterogeneity leading to poor overall survival rates worldwide. The disease is often diagnosed at an advanced stage, resulting in a dismal prognosis due to factors such as metastasis, high intra-tumor heterogeneity, and resistance to chemotherapy [2, 3]. Although the classification and terminology of gastric cancer subtypes can vary across different regions, it is widely acknowledged as a fatal disease. Improved diagnosis and precision medicine strategies are urgently needed to address the current challenges and enhance the prognosis for patients [4]. Epigenetic mechanisms involve hundreds of proteins that tightly coordinate to maintain the normal structure of the epigenome. Somatic mutations or alterations in the levels of epigenome regulatory factors play a crucial role in the formation of cancer epigenomes [5].

The AlkB gene found in E. coli codes for a DNA repair enzyme which plays a crucial role in the reversal of DNA lesions, specifically 1-methyladenine (1 mA) and 3-methylcytosine (3mC). This DNA repair enzyme is a member of the AlkB family, which is classified as an Fe (II)- and α-ketoglutarate (αKG)-dependent dioxygenase [6–8]. However, research over the years has shown that AlkB homologs are widely distributed in eukaryotic cells and function as demethylases on various substrates, including DNA, RNA, and histones [9, 10]. The mammalian AlkB family consists of nine homologs, including ALKBH1-8 and FTO, all of which possess the dioxygenase functional domain [11, 12]. Although they share similar structural features, they are found in different cellular compartments and catalyze different substrates, leading to distinct biological functions [13–16].

ALKBH1 is a member of the human AlkB family, with a protein structure consisting of 389 amino acids and a molecular structure containing a highly conserved double-stranded β-helix (DSBH) fold, which is a characteristic feature of the ⍺KG-dependent dioxygenase superfamily and a central catalytic core [16, 17]. The primary demethylating enzymatic activity of ALKBH1 is on nucleic acids such as DNA, mRNA, and tRNA [18–20]. As a DNA demethylase, ALKBH1 primarily targets N6-methyladenine (N6mA) in DNA. The N6mA motif is closely associated with the common heterochromatin marker H3K9me3, leading to alterations in chromatin accessibility, as evidenced by CHIP-seq studies [21, 22].

ALKBH1's interaction with diverse substrates highlighted the significance of genomic DNA's N6mA modification, especially in cancer. In pancreatic ductal adenocarcinoma (PDAC), the downregulation of ALKBH1 disrupted mitochondrial DNA-encoding gene transcription, triggering mitochondrial impairment. Sirtuin4, on the other hand, modulated ALKBH1 stability by deacetylating the HRD1-SEL1L complex, thereby maintaining mitochondrial equilibrium in PDAC cells [23]. Conversely, ALKBH1 was markedly upregulated in colorectal and breast cancers, correlating with metastasis and an unfavorable prognosis [24]. Intriguingly, breast cancer investigations revealed a 7% genetic alteration involving ALKBH1 [25]. In lung cancer, ALKBH1's elevated expression was linked to enhanced invasion and migration of cancer cells in vitro, while its silencing significantly curtailed these abilities. This phenomenon was substantially potentiated by ALKBH1 overexpression [25]. Similarly, a study on tongue squamous cell carcinoma (TSCC) unveiled heightened genomic N6mA levels in TSCC tissues and cultured cells. Suppression of ALKBH1 led to heightened N6mA levels in genomic DNA, promoting tumor colony formation and cell migration [26]. In ovarian cancer, the genetic alteration rate of ALKBH1 stood at 16%. In addition to lowered tumor expression levels, an overall reduction in methylation levels was also observed [27]. ALKBH1's demethylation of 6 mA inhibits NRF1-driven transcription, impacting genes in the AMPK signaling pathway. This inhibition shifts metabolism toward the Warburg effect, promoting STAD tumorigenesis [5].

This study aimed to investigate the theragnostic value of ALKBH1 in STAD using integrated analysis. We conducted differential gene expression, protein correlation, pathway, and prognostic analyses to examine the clinical significance of ALKBH1 in different tumor types and stages. Our results confirmed the clinicopathological significance of ALKBH1 in STAD patients and identified it as a potential prognostic biomarker. We also determined the association between ALKBH1 expression and TIME at the single-cell and whole-tissue levels, and identified a possible mechanism accounting for its tumor-promoting role. Additionally, our study compared ALKBH1 expression with immune-infiltrating cells and correlation of immunomodulatory factors, suggesting its potential as an immunotherapeutic target. Finally, we accessed the Cancer Drug Sensitivity Genomics (GDSC) and Cancer Cell Lineage Encyclopedia (CCLE) cell repositories, and identified ALKBH1 as a validated drug candidate in the context of targeting.

## Materials and methods

### Multi-omics analysis of gene expression differences and prognostic implications

To gain insights into gene mutations, DNA copy number alterations (CNA), gene domain variants, and mRNA expression, bioinformatics analyses were conducted using established methods [28, 29]. The Gene Expression Omnibus (GEO) database, maintained by the National Center for Biotechnology Information (NCBI), was searched using "gastric cancer" and "immune" keywords, and microarray data from all samples were extracted. DEGs were identified using Gene Expression Profiling Interactive Analysis 2 (GEPIA2) [30, 31] based on TCGA and genotypic tissue expression (GTEx) data, with a threshold of absolute fold change (FC) > 1 and adjusted p-value (adj. P) < 0.05. The DEGs of interest were further analyzed. In addition, gene variants and ALKBH1 features in STAD were analyzed by integrating the Cancer Genome Atlas (TCGA) database cBioPortal platform [32, 33]. To conduct survival analysis, we utilized the Kaplan–Meier mapper tool to investigate the association between clinical staging of STAD and several factors including immune cell content and tumor mutation load. The tool was configured to utilize an "automatic selection of optimal cutoff" function to determine the most suitable patient grouping for survival analysis.

### Analysis of single-cell transcriptomics and immune profiling

The utilization of single-cell RNA sequencing (scRNA-seq) has provided a robust approach to investigate cellular heterogeneity and gene expression at a single-cell level. We acquired scRNA-seq data from the GEO database (GSE162115 and GSE159929 datasets) and executed quality control (QC) through the R package Seurat, ensuring the inclusion of high-quality cells while reducing batch effects. To detect distinct cell subpopulations, we used uniform manifold approximation and projection (UMAP) clustering with the "BiocManager" and Gene Set Variation Analysis ("GSVA") packages in R. To annotate cell types, we compared the expression profiles of identified cell subpopulations with previously acknowledged cellular marker genes utilizing the "SingleR" package in R. Additionally, we followed the same methodology outlined in a previous study [34, 35], utilizing TIMER to investigate the association between different ALKBH1 genes' expression and the abundance of immune infiltrates in STAD. We also assessed the correlation between ALKBH1 expression and genetic markers of tumor-infiltrating immune cells.

### Processing of spatial transcriptomics data using seurat algorithm

In this study, we utilized spatial transcriptomic (ST) data obtained from a previous study (PMID: 35931863) and aggregated the UMIs in each bin100 defined point. The results were then analyzed and visualized to determine the expression levels and spatial distribution of ALKBH1, AQP1, and PECAM1. Clustering of similar ST points and dimensionality reduction were performed using RunPCA, FindNeighbors, and FindClusters functions. Cluster annotation was carried out based on H&E sections, and then cell markers were used for further annotation. Due to some clusters exhibiting high expression of multiple cell markers, we utilized the ssGSEA algorithm to score common cell types based on the average expression matrix of different clusters. This method has been found to be more effective in ST [36].

### Human STAD tissue microarrays and immunocytochemistry analysis

The study analyzed human STAD specimens using tissue microarray (TMA) slides obtained from SuperBioChips Laboratories in Seoul, Republic of Korea. The TMA slides consisted of samples of human STAD, metastatic cancer, and normal tissues. To conduct immunocytochemistry (ICC) on the TMA slides, we followed the protocol described in a previous study [37, 38]. A fluorescent multiplex staining kit (BioTnA, TATS01F, Kaohsiung, Taiwan) along with specific antibodies are utilized in labeling and detecting desired proteins in tissue samples for ICC. Samples were fixed with paraformaldehyde (4%) and permeabilized with Triton X-100 (0.2%). Non-specific antibodies were blocked with BSA (5%) before specific antibodies were applied. After incubation with primary antibodies overnight at 4 °C, secondary antibodies were applied and incubated for 30 min at room temperature. The slides were examined and analyzed by Li-Tzung Pathology Laboratory (Kaohsiung, Taiwan) and whole slide images were captured using a BX61VS® fully motorized fluorescence microscope (Olympus, Tokyo, Japan).

### Prediction of drug response based on ALKBH1 expression analysis using pharmacogenetics

We utilized two different datasets to investigate the drug sensitivity profiling based on ALKBH1 expression. The CRISPR-screen algorithm from the Genomics of Drug Sensitivity in Cancer (GDSC) data repository in Q-omics v.1.01 was used to analyze the drug sensitivity profiling, while the CCLE provided data on cellular sensitivity to pyridostatin based on ALKBH1 expression and copy number. Additionally, we developed a pharmacogenetic prediction model using the CRISPR-screen data repository for the GDSC algorithm in Q-omics v.1.0, which integrates publicly available data on mutations, gene expression, patient survival, immune scoring, drug screening, and RNAi screening from CCLE, GDSC, and DepMap databases [37, 39].

### Analyzed ALKBH1 protein levels in normal and tumor tissues using the Human Protein Atlas

The publicly accessible Human Protein Atlas serves as a valuable resource for studying the expression patterns of proteins in various human tissues and cancers. It provides comprehensive mRNA and immunohistochemistry (IHC)-based protein expression data for 20 different types of human cancer, among which is STAD. The IHC images in the database enable researchers to visualize protein expression levels in diverse cellular and tissue structures. In our study, we leveraged these images to compare the expression levels of various ALKBH1-containing protein genes in normal and STAD tissues. This approach allowed us to identify potential biomarkers and therapeutic targets for STAD, based on the differential expression patterns of these genes in the two tissue types [31].

### Statistical analysis

The statistical methods utilized in this study were based on a previously published protocol [40]. Gene expression correlation was assessed using Pearson's correlation coefficient, while t-tests or Fisher's exact tests were used for intergroup comparisons, and one-way ANOVA was utilized for intragroup comparisons. Statistical analysis was conducted using GraphPad Prism software (GraphPad Software, La Jolla, CA, USA). Significance was defined as p < 0.05.

## Results

### Genetic alterations of ALKBH1 in STAD revealed by comprehensive analysis

**Fig. 1:**
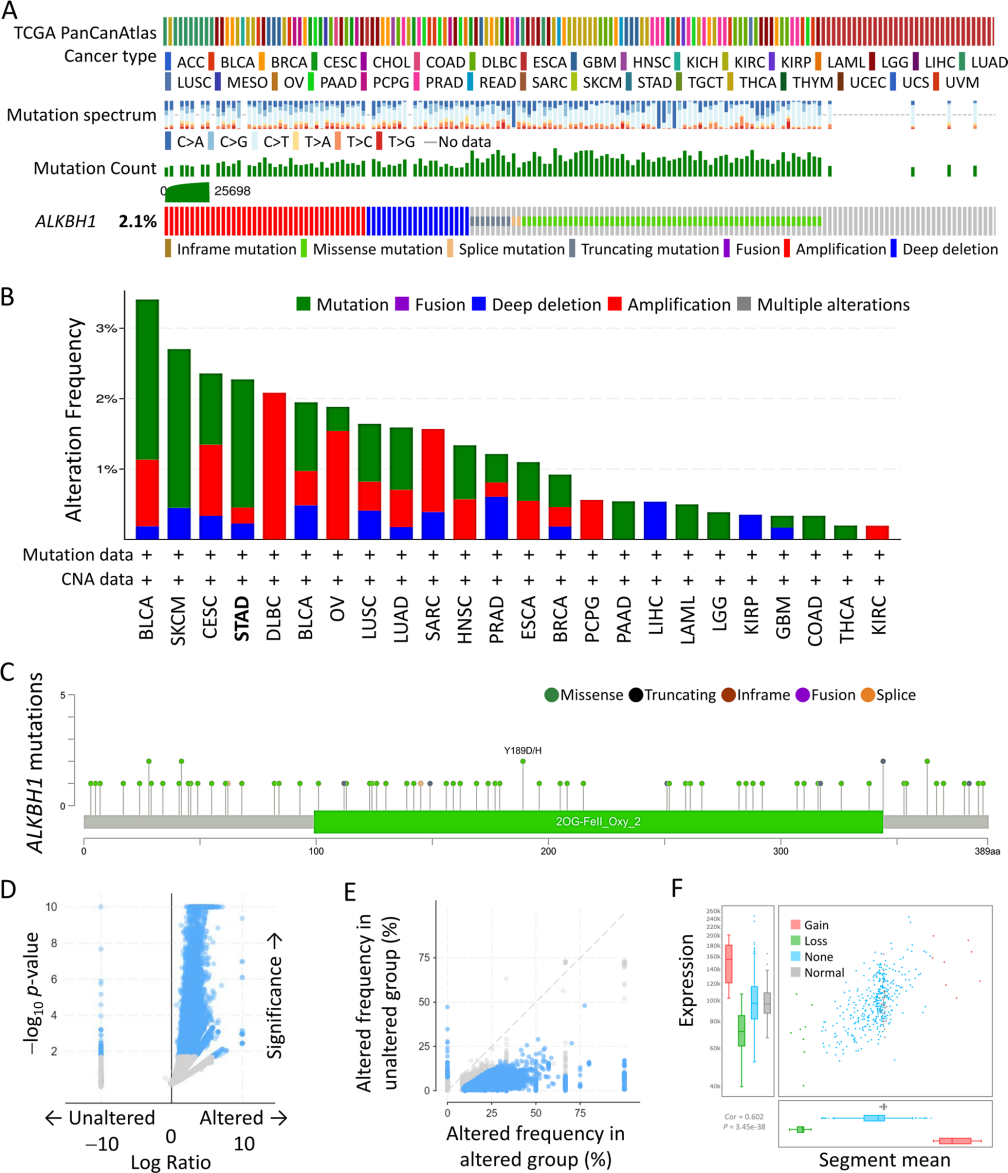
Frequency and functional enrichment analysis of ALKBH1 alterations in gastric cancer. **A** Analysis of various mutations in the ALKBH1 gene in human cancer data. **B** cBioPortal cancer genomics analysis of the frequency of ALKBH1 gene alterations in various types of cancer. **C** ALKBH1 protein domain map showing specific mutation sites. **D**, **E** Volcano and scatter plots showing frequency changes of genes associated with mutations in ALKBH1. **F** A combination of scatter and box plots to show a more detailed distribution and correlation view of copy number variation in gastric adenocarcinoma

We initially determined the frequency and type of ALKBH1 alterations in STAD, based on the STAD dataset from TCGA, and found that the ALKBH1 gene was mutated in 2.1% of all cancers by cBioPortal dataset (Fig. 1A). We further examined the genetic alterations of ALKBH1 in different tumor types in the TCGA dataset and observed that STAD tumors had the fourth highest frequency of genetic alterations, mainly mutations, Amplification, and Deep deletion (Fig. 1B). Additionally, we found that missense mutations from 389 amino acids were the primary type of genetic alteration in all TCGA tumor samples (Fig. 1C). Furthermore, our analysis of concurrent gene alteration frequencies of ALKBH1 alterations using the cBioPortal database revealed that there were 4028 samples with enriched gene alterations in both ALKBH1 altered and unaltered groups (Fig. 1D, E). Finally, we observed significant variations in the gain and loss of ALKBH1 in the copy number variation ratio distribution and boxplot (Fig. 1F).

**Fig. 2:**
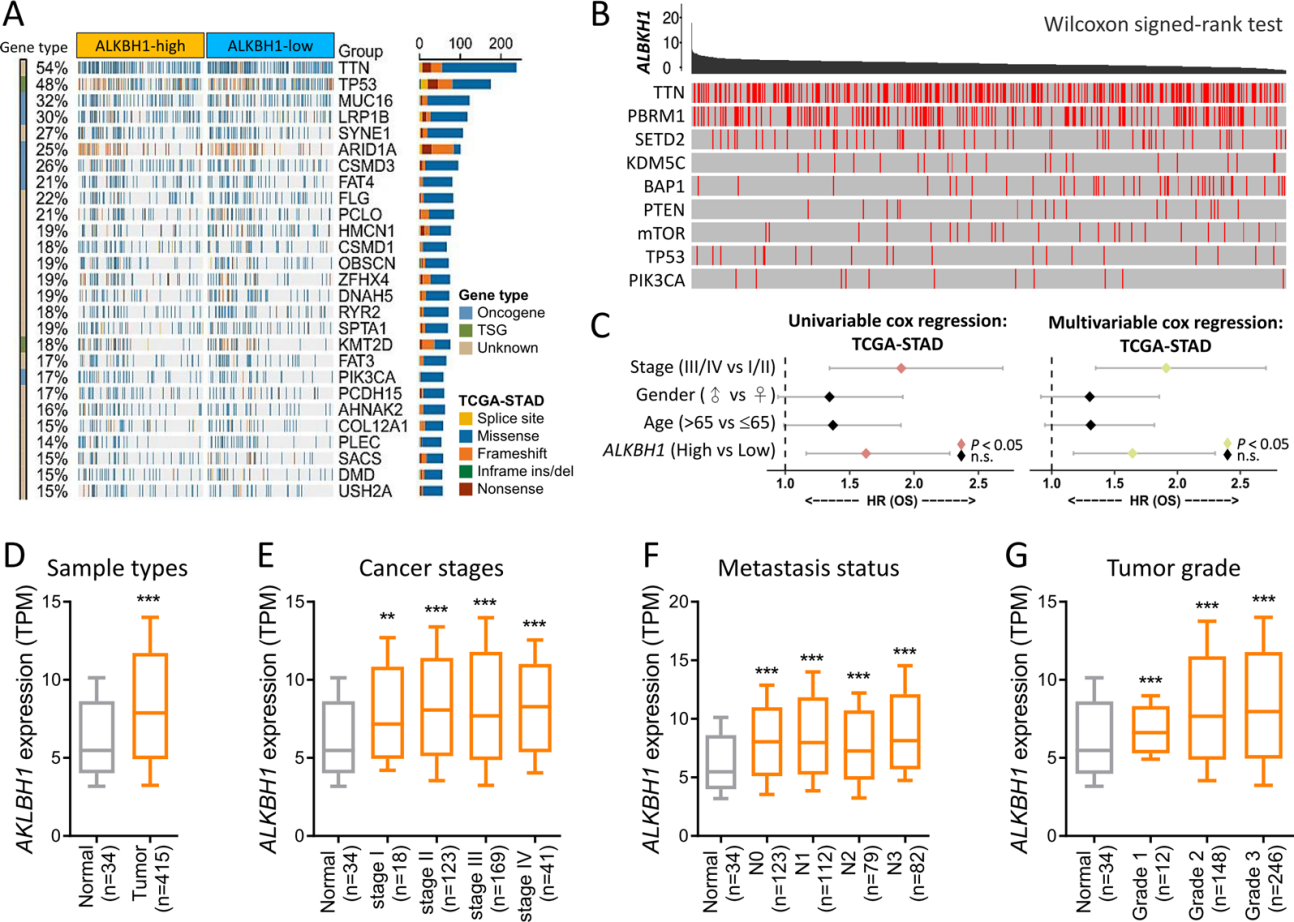
The gene landscape and expression of ALKBH1 in STAD. **A** Fisher ‘s exact test was used to compare mutation frequencies between ALKBH1-high and -low groups, with the right panel showing mutation types, driving mutation types, and groups. **B** The study investigated the relationship between ALKBH1 and the nine highly mutated genes in STAD, with red lines indicating mutation sites. **C** Univariate and multivariate Cox regression models were used to calculate the risk ratio of ALKBH1 at different stages of STAD. **D** ALKBH1 expression was compared between STAD tumor and non-tumor tissues, with box plots showing expression levels at different stages of STAD **E**, metastatic status **F** and tumor grade **G**. ** P < 0.01, *** P < 0.001 versus the normal group

We subsequently extracted data on ALKBH1 expression in STAD patients from the TCGA database and presented it using a waterfall plot that described the top 25 affected genes. Analysis of genetic variations across different ALKBH1 expression levels unveiled connections between these levels and frequently mutated genes (such as TTN, TP53, etc.) in STAD (Fig. 2A). To delve further into the impact of ALKBH1 mutations on STAD, we explored their interactions with other common genes involved in cancer progression, including TTN, PBRM1, and SETD2. In alignment with the findings in Fig. 2A, the mutated ALKBH1 gene demonstrated a more pronounced connection with TTN (Fig. 2B).

We then conducted univariate and multivariate Cox regression analyses to evaluate the relationship between ALKBH1 expression and overall survival (OS). Our findings revealed that stage, and ALKBH1 expression (high vs. low) were all significantly associated with OS. Furthermore, we performed multivariate Cox regression analysis on the same variables and discovered that risk score could act as an independent prognostic factor (*P* < 0.05) (Fig. 2C). To gain a more comprehensive understanding of ALKBH1 expression in various contexts, we examined its expression levels in different sample types, cancer stages, metastatic stages, and Helicobacter pylori infection in The Cancer Genome Atlas (TCGA) dataset (Fig. 2D–G). Our results indicate that ALKBH1 is significantly overexpressed in late stage, highly metastatic status, and highly malignant tumor grades.

### Clinicopathological significance of ALKBH1 and its prognostic value in patients

**Fig. 3:**
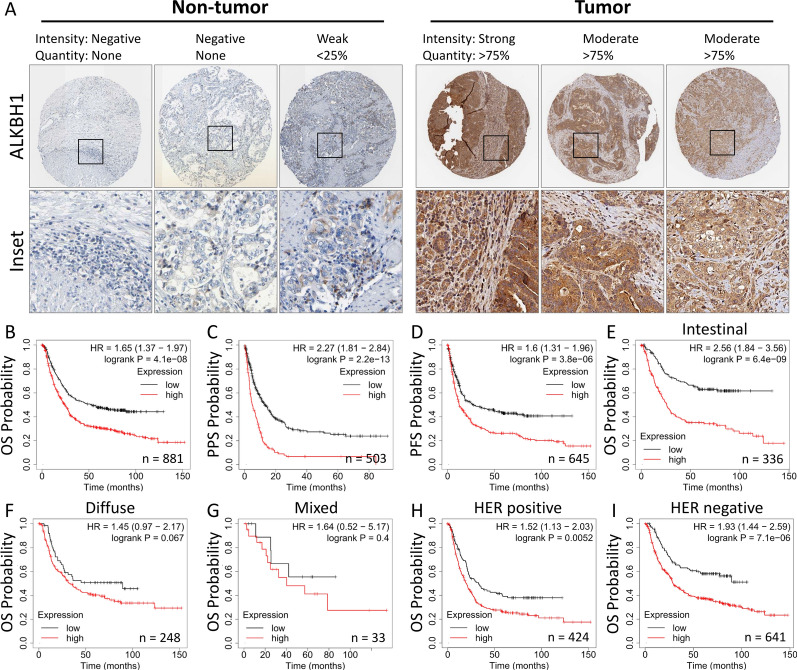
Analysis of the pathological changes of ALKBH1 in clinical gastric cancer. **A** Immunohistochemical analysis of ALKBH1 protein expression levels in STAD tissue samples from different patients based on the Human Protein Atlas. **B**–**I** Kaplan–Meier survival analysis indicating overall survival (OS) based on low/high expression of ALKBH1 **C** post-progression survival (PPS), **D** progression-free survival (PFS), **E** intestinal, **F** diffuse, **G** mixed, **H** HER + , **I** HER−. All survival analyses were based on 6 GEO datasets, including: GSE62254, GSE29272, GSE15459, GSE14210, GSE51105, GSE22377

We validated the clinical significance of ALKBH1 by analyzing immunohistochemical staining data from the Human Protein Atlas, which confirmed that ALKBH1 protein was upregulated in STAD tumor tissues compared to normal tissues (Fig. 3A). To further understand the prognostic value of ALKBH1, we performed Kaplan–Meier analysis and log-rank tests to examine the correlation between ALKBH1 expression and clinical follow-up data. We found that patients with high ALKBH1 expression had shorter overall survival (OS) time, post-progression survival (PPS), and progression-free survival (PFS) than those with low ALKBH1 expression (Fig. 3B–D). ALKBH1 was identified as an potential prognostic factor in intestinal, diffuse, and mixed types of STAD, but not in basal type (Fig. 3E–G). Additionally, ALKBH1 was found to be an independent factor in HER-positive and HER-negative patients (Fig. 3H and I).

**Fig. 4:**
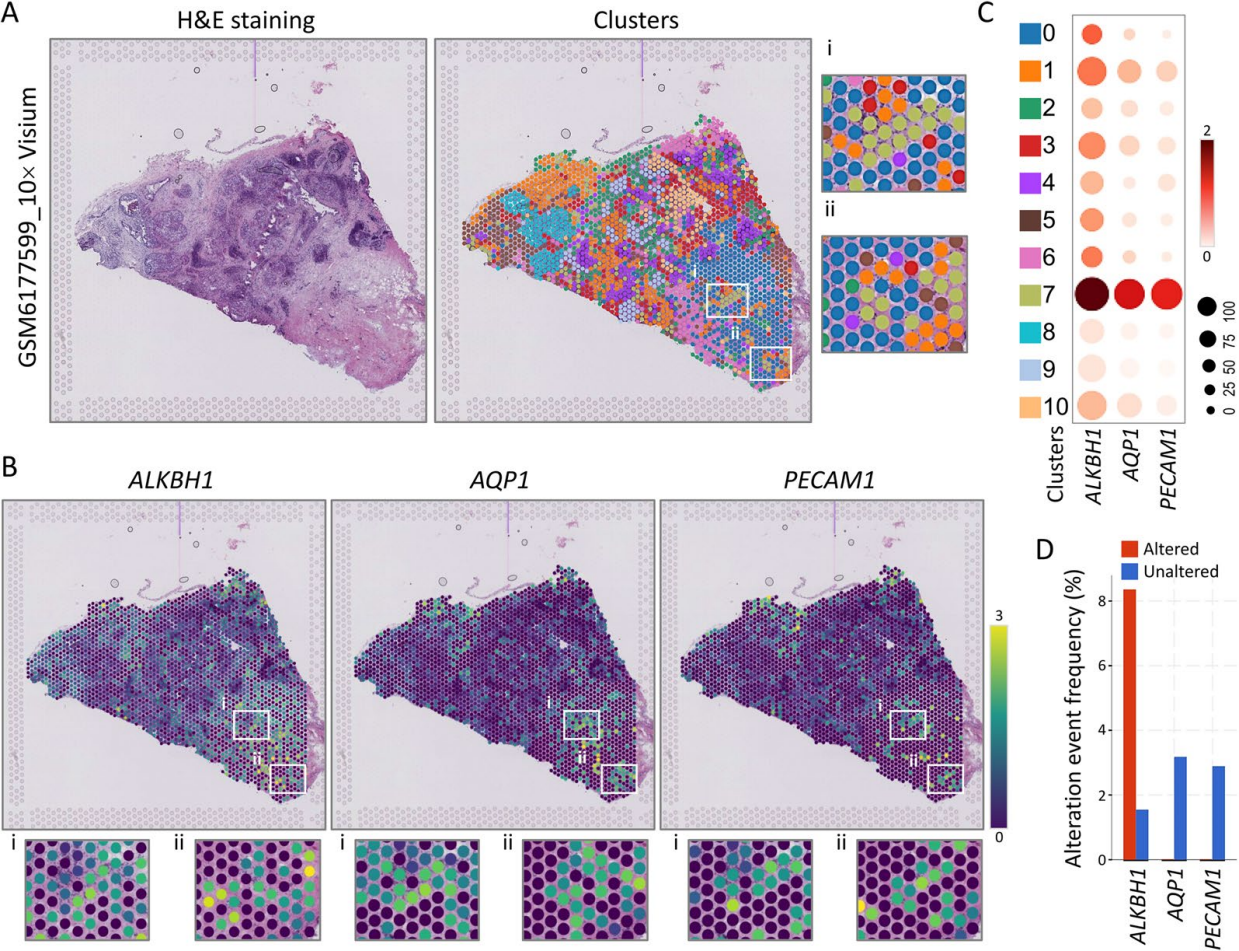
Gene expression of spatially transcriptomic-defined clusters in STAD. **A** The tissue sections were analyzed by spatial transcriptomics to identify clusters, and proper alignment with morphology was demonstrated through hematoxylin and eosin staining and cluster maps. **B** The spatial location and gene changes of ALKBH1, AQP1, and PECAM1 were visualized in different tissue sections using 10X Visium spatial gene expression. The variation of each gene in boxes i and ii was illustrated in an enlarged images. **C** A dot plot was used to show the levels of gene expression in different clusters. **D** A box plot was used to display alterations in the expression of three genes

In an effort to map transcriptomic signatures onto H&E-stained histological sections of a human reference tumor (PMID: 35931863) (Fig. 4A), we utilized ST technology. This approach involves sequencing spatially localized barcoding, which maps transcriptomic signatures directly onto histological images. Our analysis resulted in a total of 2384 counts measured, each with its own expression signature, and superimposed on the histological image of the tumor based on localized barcodes. An unsupervised clustering approach was used to group the spots according to the gene expression of each spot, with each cluster representing a specific cell type based on known marker genes and underlying histology. Space Ranger generated 10 unsupervised clusters (Fig. 4B) that were consistent with known marker genes for each tumor microenvironment cell, overlaid on histological features. We compared the biomarkers of STAD, AQP1, and PECAM1 with ALKBH1, which showed high levels in the lower right. Our analysis revealed that the spatial expression position of ALKBH1 was similar to that of the two biomarkers. Additionally, we examined the three gene expression levels in spots in high magnification regions to highlight histological features. Dot plots display the normalized, logarithmically transformed, and variance-scaled expressions of various cell clusters (y-axis) and signature genes (x-axis) in STAD snRNA-seq data (Fig. 4C). The data revealed that the expression of ALKBH1 was consistently higher than the other two gastric cancer biomarkers, AQP1 and PECAM1, across all 10 clusters. Moreover, the frequency of ALKBH1 gene alterations was significantly higher compared to AQP1 and PECAM1, as revealed by gene variation analysis (Fig. 4D).

### Analyzing single-cell RNA sequencing database to evaluate the ALKBH1 transcriptomics

**Fig. 5:**
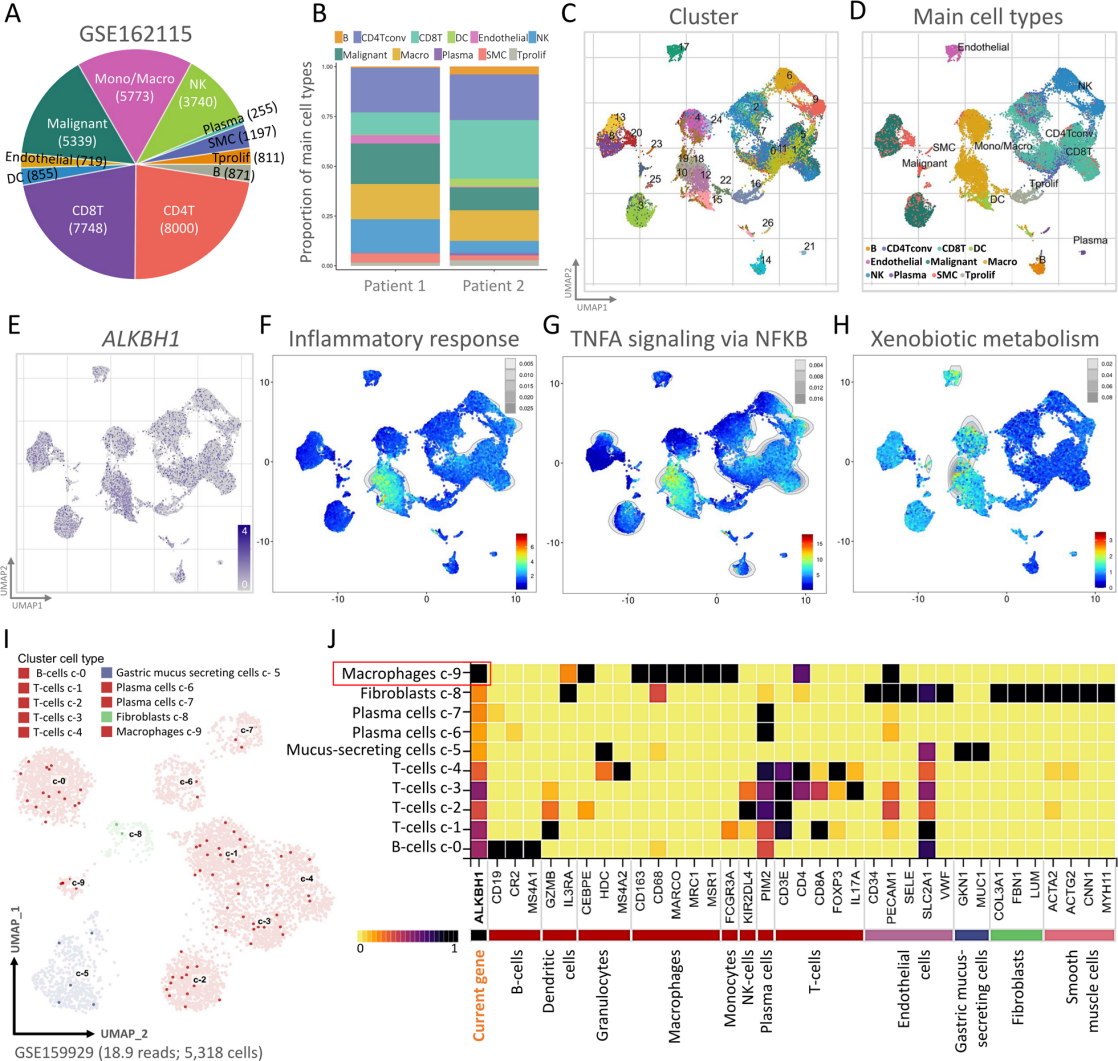
Single-cell RNA sequencing analysis enables the identification of immune cell populations. **A** and **B** Displays the relative proportions of each cell type found in the public dataset and the proportion of integrated immune cells present in the database. **C** and **D** The STAD cells from the GSE162115 dataset are visually represented using the unified flow approximation and projection (UMAP) technique and color-coded based on clusters. **E** The expression clusters of ALKBH1 are visualized using UMAP plots and subjected to gene set enrichment analysis (GSEA) **F** inflammatory response, **G** TNFA signaling via NFkB and **H** xenobiotic metabolism. **I** Visual representations of clusters of placental tissue from the GSE1599929 dataset identified using UMAP plots and bar graphs are shown through single-cell RNA sequencing. **J** The expression of the ALKBH1 gene and cell type biomarkers in different single-cell type clusters of tissues is shown in the heatmap

To evaluate the transcriptome of ALKBH1 in STAD at the single cell level and investigate the heterogeneity of various cell types in the STAD microenvironment, we conducted an analysis of the publicly available STAD single-cell RNA sequencing database (GSE162115). This database originated from two STAD patient samples and comprised of 11 cell types and 26 clusters. After removing batch effects and quality control, we analyzed a total of 35,308 cells and identified 26 significant cell populations using UMAP plots. Cell type-specific markers were identified based on top differentially expressed genes for each cluster, which were used for cell type classification. The expression and distribution of ALKBH1 in these single-cell sequencing databases were analyzed, and we found that ALKBH1 was highly expressed in the same region as the inflammatory response model. Additionally, TNFA signaling via NF-kB and xenobiotic metabolism were also generally increased, as shown in Fig. 5G, H.

The strong association between ALKBH1 overexpression and the development of STAD was demonstrated in Fig. 2E, while Fig. 5F, G suggested that ALKBH1 was linked to immune cells. Consequently, we conducted further analysis on the impact of ALKBH1 on immune cell infiltration in STAD using another publicly available database. Through a single-cell RNA sequencing dataset (GSE159929) (Fig. 5I), we examined transcriptomic data, characterized heterogeneous cell populations, and investigated changes in immune cells in the tumor microenvironment. We obtained UMAP maps of 5,318 single cells and analyzed the specificity and distribution of ALKBH1 in different cell populations of STAD to determine the gene expression variation in each cell type. The heatmap revealed that ALKBH1 was highly expressed in gastric tissues, where the biomarkers of five macrophages also exhibited high expression levels (Fig. 5J).

### Association of macrophage infiltration with overexpression of ALKBH1

**Fig. 6:**
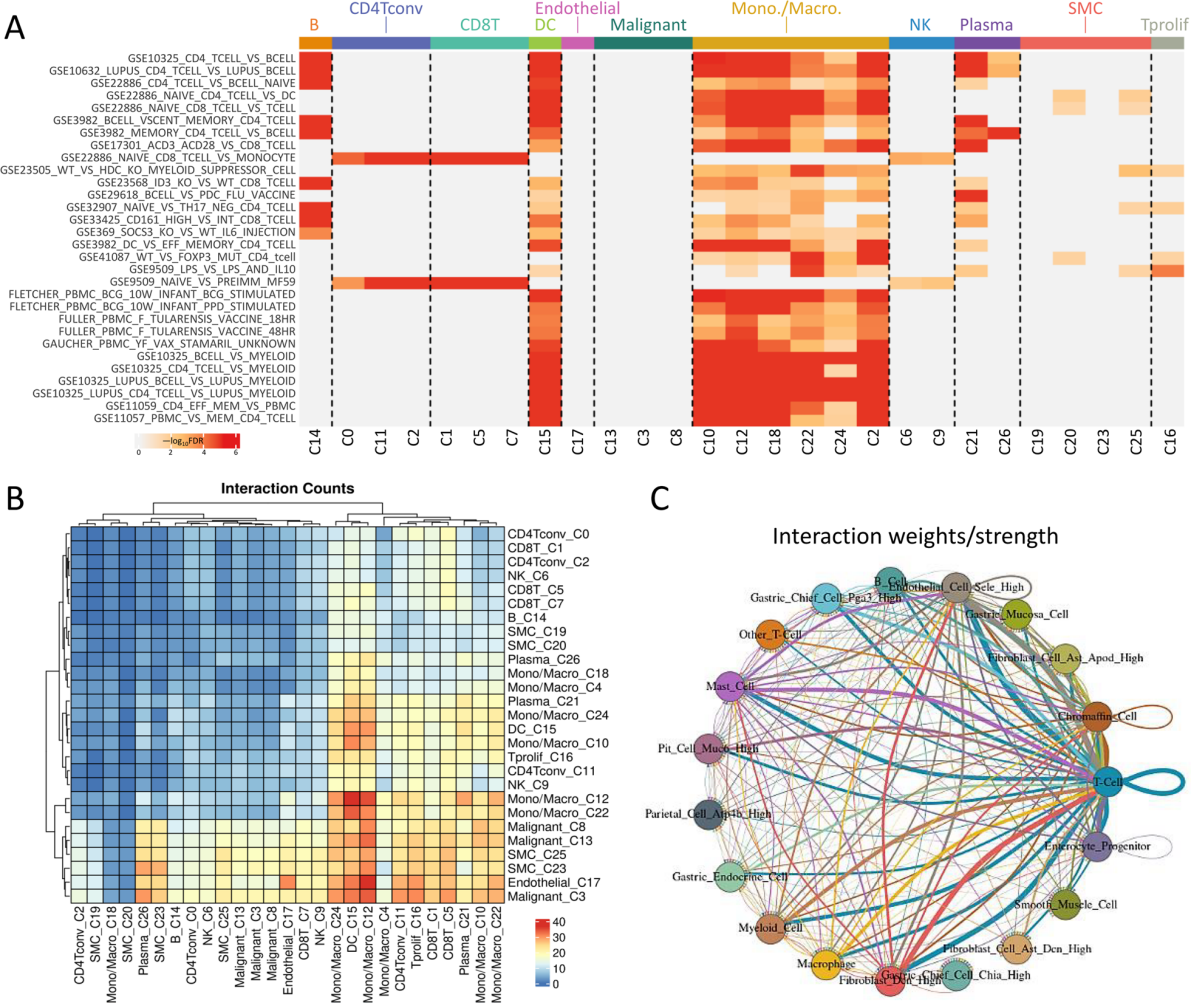
Investigation of the impact of ALKBH1 on the tumor immune microenvironment (TIME). **A** Immunological analysis of the immune infiltrate was conducted using the GSE162115 database, as illustrated. Moreover, a heat map was generated to demonstrate the association between ALKBH1 expression and immune infiltration in human gastric cancer cells. **B** A heat map is presented to exhibit the correlation between ALKBH1 expression and lymphocyte infiltration in human cancers. **C** A circular plot was constructed to visualize the potential ligand-receptor interactions, where the edge's width represents the strength of the interaction

In this study, we investigated the potential correlation between ALKBH1 and STAD by performing gene set enrichment analysis (GSEA) using differentially expressed genes (DEGs). We identified clusters highly associated with macrophages from the single-cell RNA-sequencing database shown in Fig. 5A–H. Since macrophages exhibit tissue-resident properties and possess pro- or anti-inflammatory functions, we further analyzed the relationship between ALKBH1 and tumor immune interactions (Fig. 6A). Given the critical role of the tumor immune microenvironment (TIME) in tumor progression, metastasis, and immune evasion, we conducted Spearman correlation analysis by TISIDB, which revealed a positive correlation between ALKBH1 expression and the majority of tumor-infiltrating macrophages in STAD (Fig. 6B). Moreover, we observed complex interactions between various T cell subpopulations and macrophages, which were the strongest intercellular communication detected in STAD, as retrieved from the scTIME portal (Fig. 6C).

**Fig. 7:**
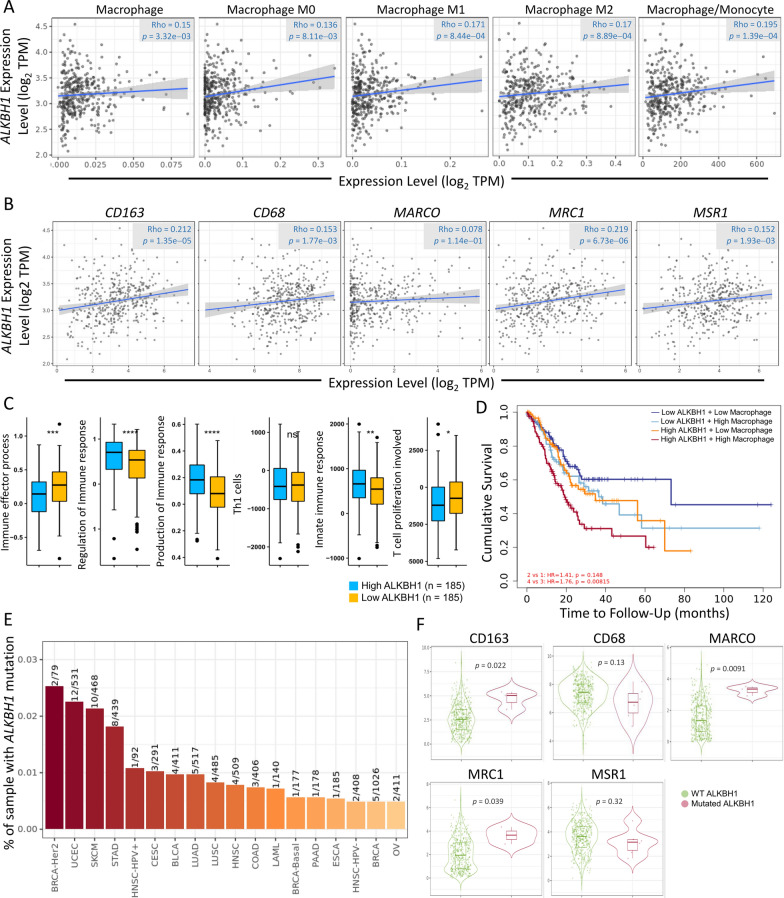
Exploring the link between ALKBH1 and immune infiltration in STAD. **A** The relationship between ALKBH1 levels and various macrophage subtypes was explored. **B** The correlation between ALKBH1 and genes related to macrophages was investigated. **C** Effect of different ALKBH1 expression on immune cells. **D** The Kaplan–Meier plots of macrophage and ALKBH1 mRNA expression to visualize the survival differences in STAD. **E** The Gene_Mutation module assesses the incidence of ALKBH1 gene mutations across multiple cancer types. Examples of mutational profiles and immunogenicity analyses are presented. **F** The analysis of the association between ALKBH1 mutations and macrophages

We aimed to investigate the potential association between ALKBH1 expression and the tumor microenvironment of STAD, particularly in macrophages. We assessed the correlation between ALKBH1 and M0, M1, and M2 macrophage subtypes, and found a positive correlation (Fig. 7A). Furthermore, we examined macrophage biomarkers and observed a positive correlation between ALKBH1 and CD163, CD68, MARCO, MRC1, and MSR1 (Fig. 7B). To validate the association between ALKBH1 and immune cells, we compared low and high ALKBH1 expression groups in terms of immune-related functions. The difference between the low and high ALKBH1 groups was significant for immune effector process, regulation of immune response, production of immune response, innate immune response, T cell proliferation involved, but not for "Th1 cells" (Fig. 7C). Patients with high ALKBH1 expression and high macrophage infiltration exhibited shorter survival times than those with high gene expression only (Fig. 7D). We investigated whether ALKBH1 mutations also affect macrophages. Using the mutation module in Pan-cancer, we analyzed the effect of ALKBH1 mutations on immune cell infiltration in various cancer types. Our results showed that ALKBH1 mutations were the fourth most frequent in pan-cancer (Fig. 7E). We also examined the correlation between ALKBH1 mutations and macrophage biomarkers mentioned earlier. We found that the expression of CD163, MARCO, and MRC1 was significantly elevated in mutated ALKBH1, while CD68 and MSR1 showed no significant difference (Fig. 7F).

**Fig. 8:**
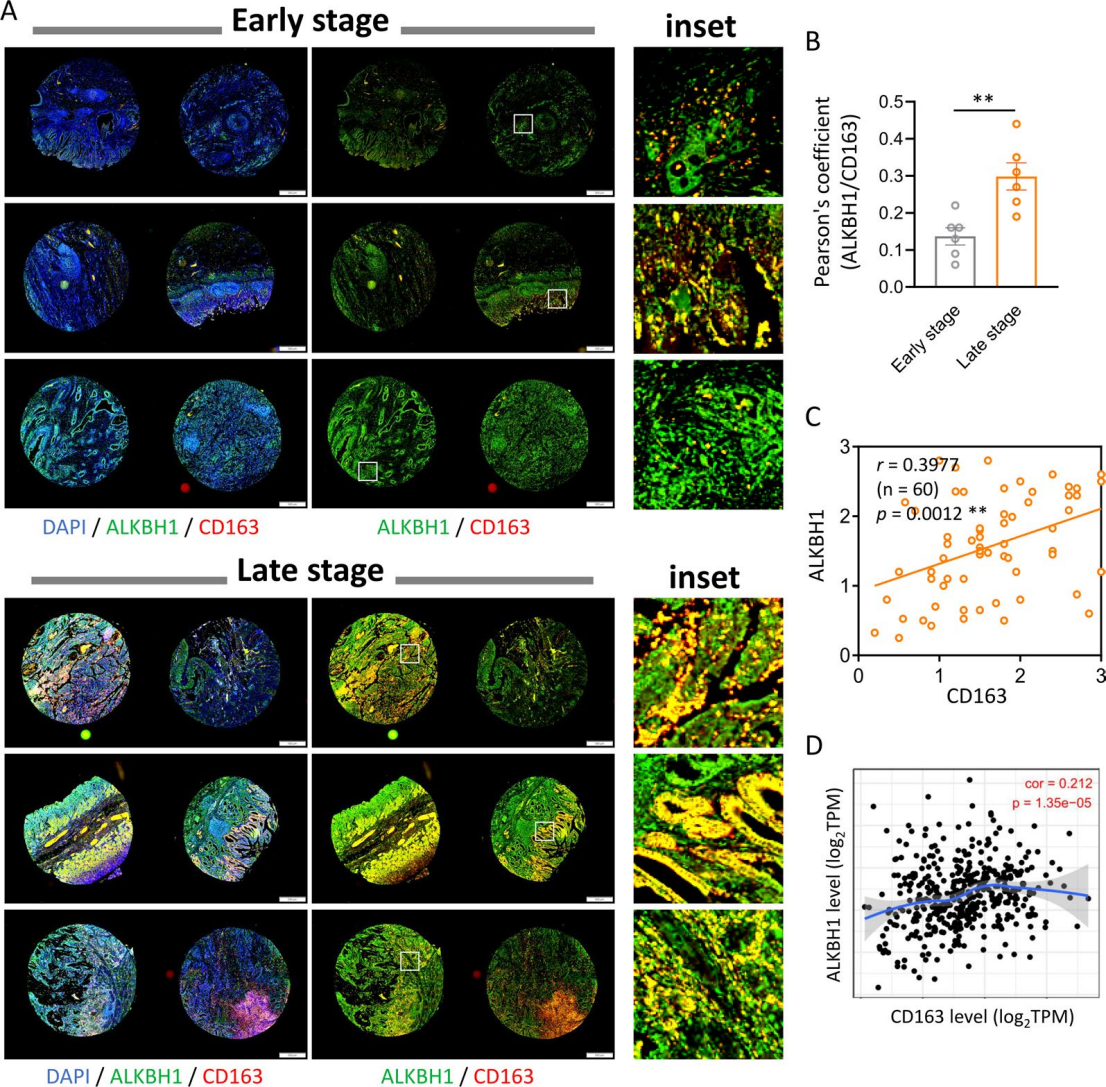
Investigation of the correlation between ALKBH1 and CD163 expression in tumor biopsies during STAD progression. **A** Whole TMA was triple-labeled with immunofluorescence of ALKBH1, CD163 and DAPI, followed by panoramic tissue scanning. **B** The Pearson's correlation coefficient was used to display the overlapping values of ALKBH1 and CD163 fluorescence signals. **C** A scatterplot was used to illustrate the normalized Spearman correlation between ALKBH1 and CD163 for each patient reading. **D** The correlation between ALKBH1 and CD163 expressions in STAD from the CIBERSORT dataset was shown in a scatter plot. ** p < 0.01

In order to validate the relationship between ALKBH1 and macrophages in STAD, we conducted a triple-labeling immunofluorescence study of ALKBH1, CD163, and DAPI on the entire STAD in TMA. The STAD TMA samples were divided into early and late stages, and their fluorescence intensities were analyzed using panoramic tissue scanning. The high intensity of green fluorescence ALKBH1 and red fluorescence CD163 in the late-stage group can be observed in Fig. 8A, with both red and green fluorescence consistently increased. The magnified image clearly shows a large amount of ALKBH1 in gastric cancer tissues, with advanced tumors showing a large amount of red color, resulting in some tissues appearing yellow. After quantifying the fluorescence signals, we confirmed a positive correlation between ALKBH1 and CD163 (Fig. 8B). We also analyzed the signals of ALKBH1 and CD163 in each of the 60 biopsies and confirmed their positive correlation (Fig. 8C). Additionally, we analyzed the correlation between ALKBH1 and CD163 using the TMIER database, and the results were similar to those presented in Fig. 8D.

### Examination of the clinical and pathological importance of ALKBH1 in STAD from a pan-cancer perspective

**Fig. 9:**
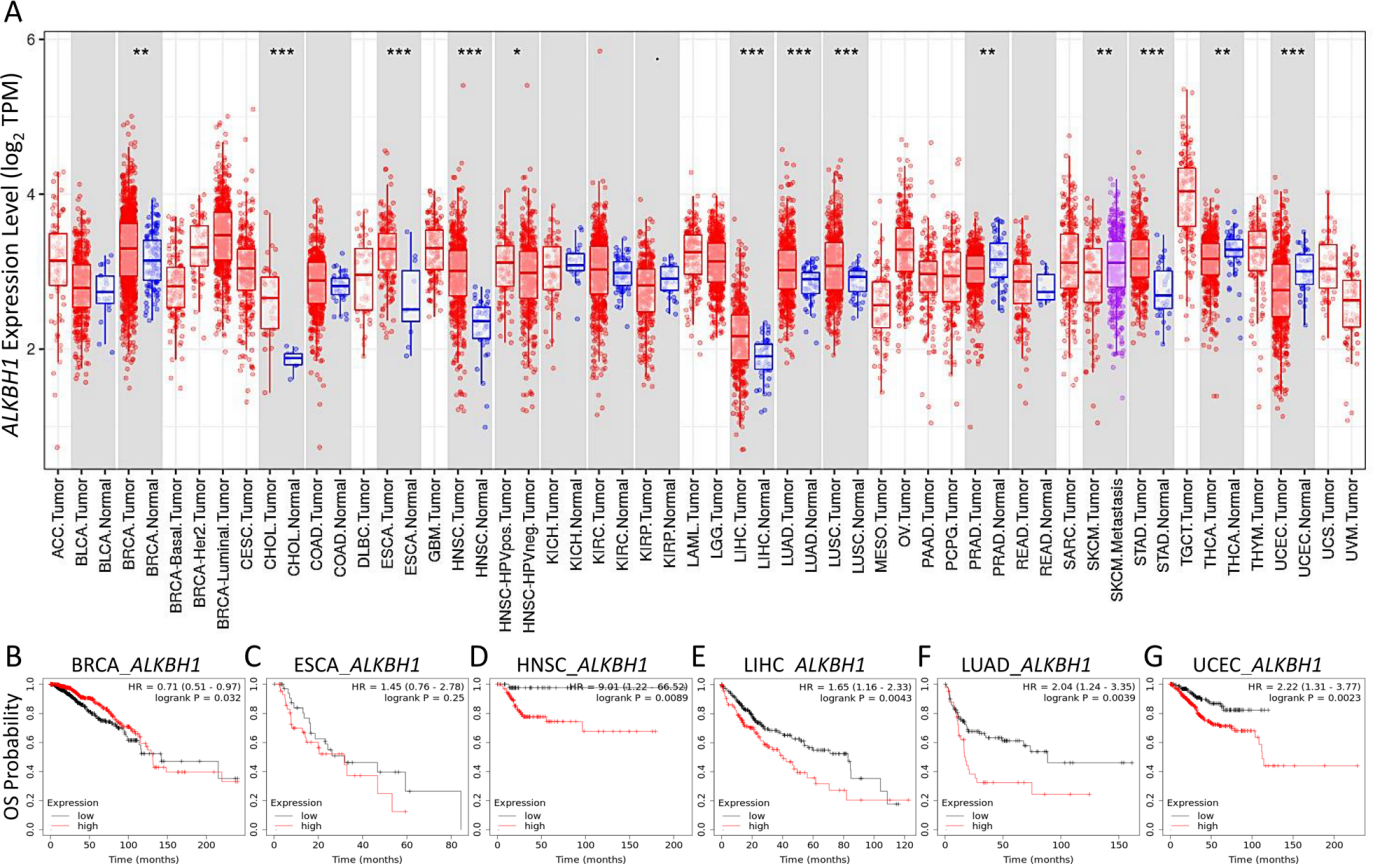
Bioinformatic validation of the clinical significance of ALKBH1 across multiple cancer types. The clinical significance of ALKBH1 from a pan-cancer perspective. Expression levels of ALKBH1 in various cancers and comparison between tumor and normal tissues with statistical significance denoted by asterisks (**A**). Kaplan–Meier survival analysis of low/high ALKBH1 expression groups in BRCA (**B**), ESCA (**C**), HNSC (**D**), LIHC (**E**), LUAD (**F** and UCEC (**G**). ***p < 0.0001, **p < 0.01, *p < 0.05 between tumor and normal

To further investigate the potential clinical significance of ALKBH1, we examined its expression levels in various types of cancer and evaluated its prognostic value. They found that several types of cancer, including breast cancer (BRCA), cholangiocarcinoma (CHOL), esophageal carcinoma (ESCA), head-neck squamous cell carcinoma (HNSC), liver hepatocellular carcinoma (LIHC), lung adenocarcinoma (LUAD), lung squamous cell carcinoma (LUSC), prostate adenocarcinoma (PRAD), thyroid carcinoma (THCA), and uterine corpus endometrial carcinoma (UCEC), exhibited similar expression patterns to those observed in STAD (as shown in Fig. 9A). Next, the we performed Kaplan–Meier analysis to assess the association between ALKBH1 expression and overall survival (OS) in each of these cancer types. Their analysis revealed that high expression of ALKBH1 was significantly associated with shorter OS time in BRCA (as shown in Fig. 9B), ESCA (Fig. 9C), HNSC (Fig. 9D), LIHC (Fig. 9E), LUAD (Fig. 9F), and UCEC (Fig. 9G). These findings suggest that ALKBH1 may have a broad clinical value in predicting prognosis across multiple cancer types.

### Identification of potential ALKBH1 inhibitors for STAD through pharmacogenetic screening

**Fig. 10:**
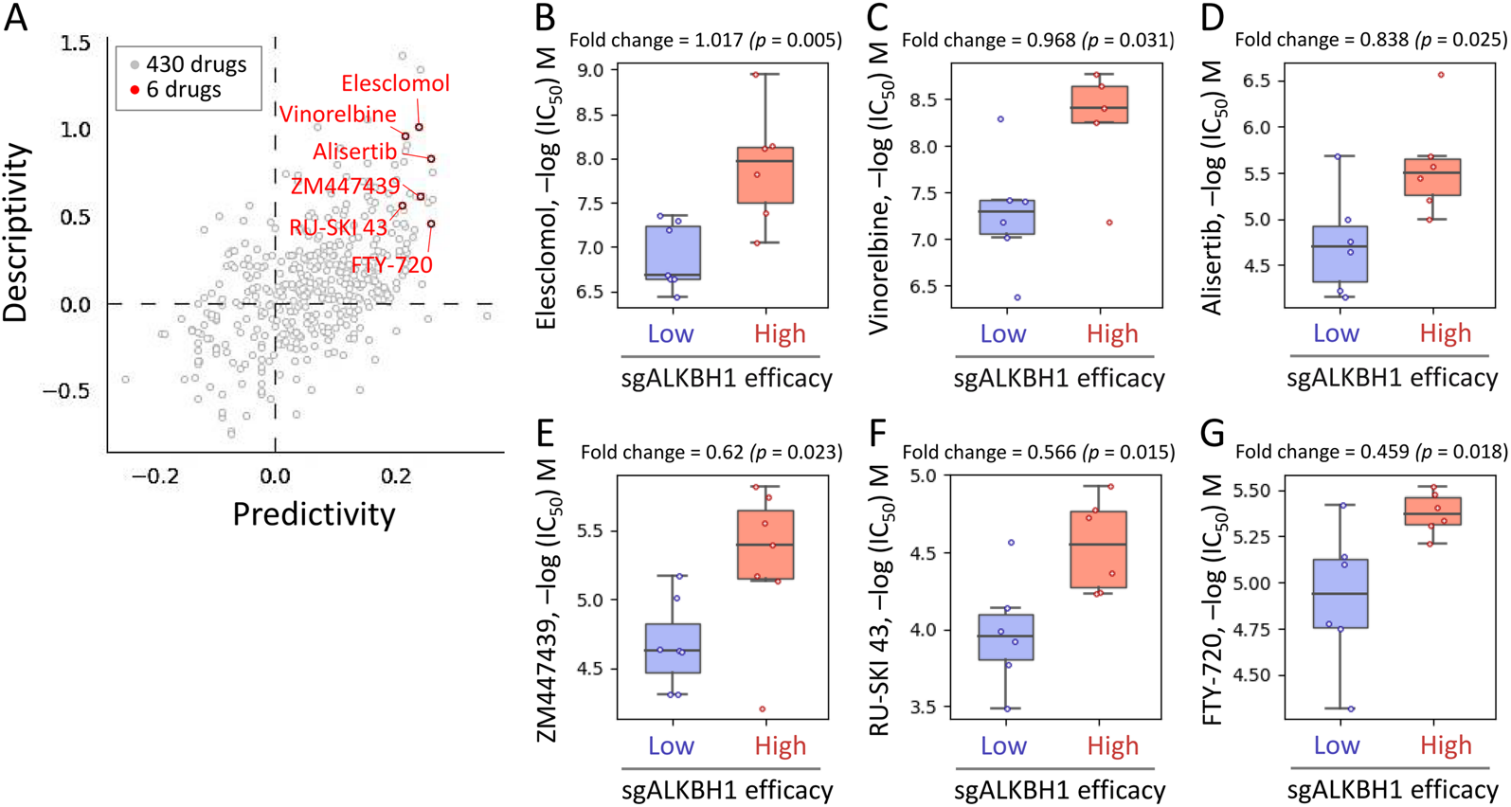
Analysis of drug sensitivity and cytotoxicity in gastric cancer cells. **A** The scatter plot illustrates the cross-association scores of predictivity and descriptivity that were used to identify potent drugs with efficacy against STAD cells. To identify gene signatures and potential drugs, we queried the pharmacogenetics database for the ALKBH1 gene. We then evaluated the drug sensitivity of the sgALKBH1 gene to various chemical drugs in STAD cell lines. The boxplots **B**–**G** depict the log of the half maximal inhibitory concentration (IC50) values for six drugs, namely Elesclomol, Vinorelbine, Alisertib, ZM447439, RU-SKI 43, and FTY-720, that showed altered potency

The study aimed to identify potential drugs with efficacy against STAD by examining the GDSC database for drugs that displayed increased potency in the presence of high ALKBH1 expression. We conducted cross-association analyses between drug response and the CRSPR knockout of ALKBH1 using single-guide RNA (sgRNA) in different STAD cells. Our performed cross-correlation analyses to investigate the effects of 430 drugs on sgRNA-mediated ALKBH1 in various STAD cells (Fig. 10A). The analysis identified six small molecule drugs, namely Elesclomol, Vinorelbine, Alisertib, ZM447439, RU-SKI 43, and FTY-720, that displayed altered potency (Fig. 10B–G). The study found that STAD cell lines with high sgALKBH1 efficiency were more sensitive to these drugs. Overall, the results suggest that these drugs have the potential to be used as anticancer agents targeting ALKBH1 to regulate gastric cancer cell growth.

## Discussion

ALKBH1 is a multifaceted enzyme that has been implicated in various biological processes, including DNA and RNA demethylation. Among its substrates, N6-methyladenosine (N6mA) on genomic DNA is considered the most critical for cancer effects [41]. This study highlights that Copy Number Variations (CNV) and mutation status associated with the ALKBH1 gene regulate the progression of STAD, concurrently influencing immune cell infiltration in the tumor microenvironment. We will comprehensively explore the roles played by ALKBH1 gene variations in various cancers. In glioblastoma, N6mA levels are significantly elevated and co-localized with heterochromatin histone modifications, mainly H3K9me3. Downregulation of ALKBH1 leads to increased levels of N6mA in genomic DNA, which coordinates with H3K9me3 to reduce chromatin accessibility and silence transcription of some oncogenes [18]. In a patient-derived human glioblastoma model, the deliberate reduction of ALKBH1 expression through targeted knockdown proved to be highly effective. This intervention resulted in a substantial inhibition of tumor cell proliferation, subsequently leading to a significant extension in the survival of mice involved in the study. These findings hold promise for the development of novel therapeutic strategies in the context of glioblastoma treatment [21]. However, the function of ALKBH1 in cancer seems to vary depending on the tissue type. For instance, in squamous cell carcinoma of the tongue (TSCC), both TSCC tissues and cultured cells exhibit heightened levels of genomic N6mA [17]. Surprisingly, silencing ALKBH1 increases N6mA levels in genomic DNA, ultimately amplifying tumor colony formation and cell migration [42]. In contrast, targeted knockouts of N6AMT1 and METTL4, two methyltransferases that typically counteract the effects of ALKBH1, significantly reduced genomic N6mA levels. This reduction markedly inhibited the ability of TSCC cells to form colonies and migrate. These findings underscored the complex regulatory network involving these methyltransferases and suggested their potential as therapeutic targets for TSCC [13].

On the other hand, the knockdown of N6AMT1 and METTL4, which are methyltransferases with opposing functions to ALKBH1, results in a reduction of genomic N6mA levels, leading to the inhibition of TSCC cell colony formation and cell migration [26]. Similar results were observed in the study of HCC, where the overexpression of N6AMT1, a methyltransferase, increased N6mA levels, resulting in enhanced cell viability, reduced apoptosis, and increased cell migration and invasion, whereas the overexpression of ALKBH1 had the opposite effect [43]. Additionally, overexpression of ALKBH1 was found to reverse the inhibitory effect of miRNA-339-5p, an upstream gene of ALKBH1, on the migration and proliferation of STAD [44]. Apart from its role as a genomic DNA N6mA demethylase, ALKBH1 can also bind mRNA and act as a demethylase. In both lung cancer tissues and cultured cells, there was an observed upregulation in the expression of ALKBH1. Silencing ALKBH1 in these lung cancer cells led to a significant inhibition of their in vitro invasion and migration capabilities. Conversely, when ALKBH1 was overexpressed, it notably enhanced these invasive and migratory properties. These findings shed light on the critical role of ALKBH1 in lung cancer progression and its potential as a therapeutic target [45]. Similarly, ALKBH1 overexpression promotes metastasis in CRC through modifying METTL3 mRNA m1A levels, resulting in reduced protein translation, increased m6A demethylation of SMAD7 mRNA, and enhanced tumor migration and invasion. Silencing SMAD7 can significantly reverse cell migration and invasion defects caused by depletion of ALKBH1 and METTLE3 [24].

The present study investigates the potential of ALKBH1 as a diagnostic and prognostic biomarker for immune infiltration in STAD. It is well established that the tumor microenvironment and immune cell infiltration play crucial roles in the host's immune response to cancer cells. In this research, we have showcased the power of single-cell RNA sequencing (RNA-seq) technology, which facilitates the profiling of transcriptomes at the individual cell level. This cutting-edge technology offers unprecedented capabilities to dissect intricate aspects of tumorigenesis and cancer progression. By employing these advanced techniques, we can precisely sequence transcripts, thereby enhancing our comprehension of the diverse cellular composition within the STAD tumor microenvironment. Furthermore, it allows us to unravel the intricate interactions among cells within the complex and heterogeneous landscape of cancer tissues. Our findings suggest that the ALKBH1 cluster network is involved in inflammatory and immune-related pathways and may serve as a new diagnostic window for monitoring the tumor microenvironment. We validated our hypothesis using a multicomponent approach that analyzed data from TCGA, spatial transcription from GEO database, and single-cell sequencing datasets. Our analysis revealed a positive correlation between macrophage infiltration and ALKBH1 expression in the STAD infiltration cohort, suggesting that ALKBH1 may reflect immune status in addition to disease prognosis. The pathway enrichment analysis of the ALKBH1 positive and negative correlation clustering network supports this observation. Thus, these findings suggest that ALKBH1 is involved in the immune infiltration of STAD and may serve as a prognostic biomarker of the immune response to these cancers. Importantly, our study provides clinical implications for the prognostic evaluation and follow-up management of immunotherapy.

This research is not without its limitations. We conducted a drug screening process, explored various cell lines using pharmacogenomics, and identified six potential targets for ALKBH1 inhibition in STAD cells. Furthermore, there is a need to recruit a substantial number of patients with STAD for the examination of ALKBH1 and the analysis of its correlation with tumor progression. While targeting the ALKBH1 signaling axis could potentially yield dual benefits by suppressing genes and enhancing the immunotherapeutic response in STAD, it's important to note that further experimental validation is required to delve into the molecular mechanisms linked to ALKBH1 in STAD. In summary, our study contributes to a deeper comprehension of ALKBH1's role in STAD, particularly from the standpoint of clinical tumor samples, and paves the way for the exploration of novel immunotherapeutic strategies.

## Conclusion

**Fig. 11:**
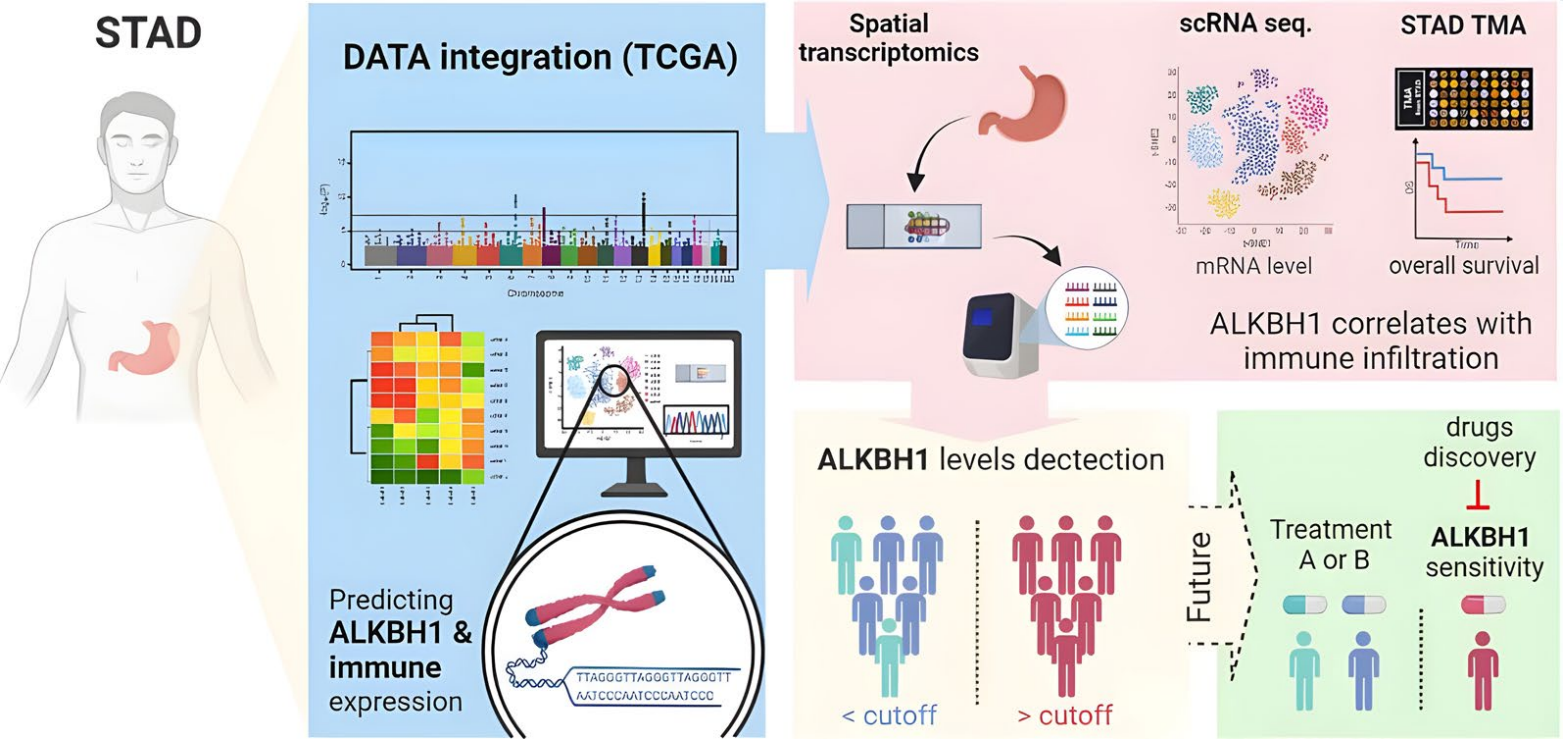
The model illustrates how ALKBH1 may have significant implications in various areas of STAD, including diagnosis, prognosis, tumor immune microenvironment, and precision treatments

In summary, this study unveils crucial insights into the intricate molecular biology of ALKBH1, particularly in the context of STAD. Elevated ALKBH1 expression is associated with unfavorable outcomes in STAD, impacting both the tumor microenvironment and macrophage infiltration. ALKBH1 stands out as a promising candidate for diagnostic, prognostic, and immune-related therapeutic interventions in STAD (Fig. 11). However, further investigations are necessary to validate our findings and unravel the immunomodulatory role and underlying mechanisms of ALKBH1 in STAD. Concurrently, the six drugs identified through pharmacogenomics predictions show potential for inhibiting STAD development. This study underscores the significant correlation between ALKBH1 and macrophages, pointing towards genes that merit deeper exploration for advancing diagnostic precision, treatment strategies, and prognostication for STAD patients.

## Data Availability

Not applicable.
